# Membrane thinning for efficient CO_2_ capture

**DOI:** 10.1080/14686996.2017.1386531

**Published:** 2017-10-30

**Authors:** Roman Selyanchyn, Shigenori Fujikawa

**Affiliations:** ^a^ WPI International Institute for Carbon-Neutral Energy Research (WPI-I2CNER), Kyushu University, Fukuoka, Japan; ^b^ Center for Molecular Systems (CMS), Kyushu University, Fukuoka, Japan

**Keywords:** Nanomembrane, carbon neutral energy, membrane gas separation, freestanding nanomembrane, molecularly tailored nanochannel, carbon dioxide, 50 Energy Materials, 100 Materials, 102 Porous / Nanoporous / Nanostructured materials, 105 Low-Dimension (1D/2D) materials, 306 Thin film / Coatings, 206 Energy conversion / transport / storage / recovery

## Abstract

Enhancing the fluxes in gas separation membranes is required for utilizing the membranes on a mass scale for CO_2_ capture. Membrane thinning is one of the most promising approaches to achieve high fluxes. In addition, sophisticated molecular transport across membranes can boost gas separation performance. In this review, we attempt to summarize the current state of CO_2_ separation membranes, especially from the viewpoint of thinning the selective layers and the membrane itself. The gas permeation behavior of membranes with ultimate thicknesses and their future directions are discussed.

## Graphical abstract

**Figure:**
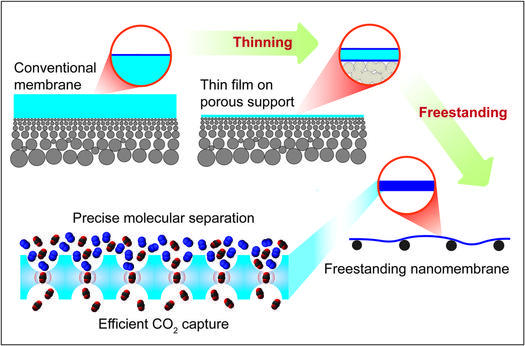

## 1. Introduction

The consequent emission of huge amounts of CO_2_ into the atmosphere after burning fossil fuels has been recognized as the main contributor to global warming and climate change [1]. As demonstrated by accurate atmospheric CO_2_ concentration monitoring at the Keeling Laboratory in Mauna Loa, Hawaii [2], and many other sites, CO_2_ concentration has undergone a steady increase every year since records began in 1958. With the latest readings, it has exceeded 400 ppm, which is the highest value recorded throughout the whole range of available data. The Transnational Paris Agreement signed in 2016 set the goal for governments to limit the further emission of CO_2_ into the atmosphere in order to reduce interference with Earth’s climate system, which is essential for sustainable food production and economic development [3].

Carbon capture and storage (CCS) is a set of technologies used to capture CO_2_ followed by its sequestration underground. This process has been introduced to massive CO_2_ emission points, such as thermal power plants and cement plants [4]. However, CCS still remains in the demonstration stage and is far from being widely and practically used worldwide. The main reason for the slow industrial deployment is the high cost of its implementation. Different techno-economic estimations of the whole process show that the CO_2_ capture process is the most cost intensive part of the whole cycle, taking up to 80% of the total capital cost [4,5]. Therefore, scientific advancements in CO_2_ capture are needed in order to increase the affordability of the whole CCS cycle and to enable its future application on a large scale [6].

The main existing approach for separating CO_2_ at mass emission points is to scrub it from the flue gas using liquid monoethanolamine (MEA), which absorbs CO_2_ at ambient temperature, followed by releasing the CO_2_ from the solution by heating. Although this is a reliable and well-optimized technology, it is less flexible for cost reduction. Additionally, the high temperatures used for CO_2_ desorption cause the degradation of amines [7].

Alternatively, the use of solid adsorbents is now being widely considered. Among the various promising materials, the most promising adsorbents are zeolites, activated carbons, calcium oxides, hydrotalcites, organic-inorganic hybrids, and metal-organic frameworks [8]. Solid adsorbents are generally required to have fast adsorption and desorption kinetics, large adsorption capacity, infinite regenerability and stability, and a wide range of operating conditions. However, in reality, all practical adsorbents have drawbacks, such as capacity degradation, slow adsorption kinetics, or instabilities in realistic conditions. Within the various supported organic adsorbents, covalently tethered amines have shown the potential to be outstanding adsorbents in cases where the adsorption capacity and adsorption kinetics can be productively balanced [8].

Carbon dioxide separation by membranes is considered one of the promising options for carbon capture [9]. The advantages of membranes over conventional and well-established liquid and solid sorbents are their small environmental footprint, energy efficiency, simplicity, manufacturing scalability, and potentially much lower economic costs [10,11]. However, currently existing membranes have not reached the stage that satisfies the requirements for treating huge amounts of flue gases emitted at, for example, conventional coal power plants. The flux of gases through membranes needs to be sufficiently improved for mass scale applications. Additionally, in order to compete with the amine-based technologies, high selectivities (~100) are required from single-stage separation systems [12].

The flux (*J*, volume of gas *V*, permeated through the membrane with area *S* during time *t*) and permeance (*P*, transmembrane pressure normalized flux) of the gas through a membrane with thickness *l* and permeability Π are provided in Equations 1 and 2, respectively(1)
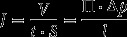

(2)
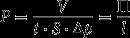



where Δp is the transmembrane pressure difference of the gas. As the permeability is considered to be a constant property of the material, it is obvious that the thickness should be minimized in order to maximize the flux or permeance through the membrane. The most common units for permeability and permeance are barrer and gas permeance units (GPU), respectively, and their following relations in SI units are provided in Equations 3 and 4


(3)
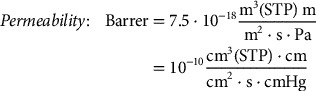

(4)
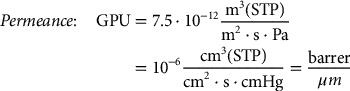



where m^3^(STP) and cm^3^(STP) are the amount of gas in m^3^ or cm^3^ at a standard temperature and pressure (273 K, 1 bar).

The ideal selectivity α_A/B_ is a parameter used to characterize the ability of a material to separate two gases A and B, and it is obtained by taking the ratio of the permeabilities of the two gases(5)
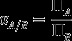



**Figure 1.**
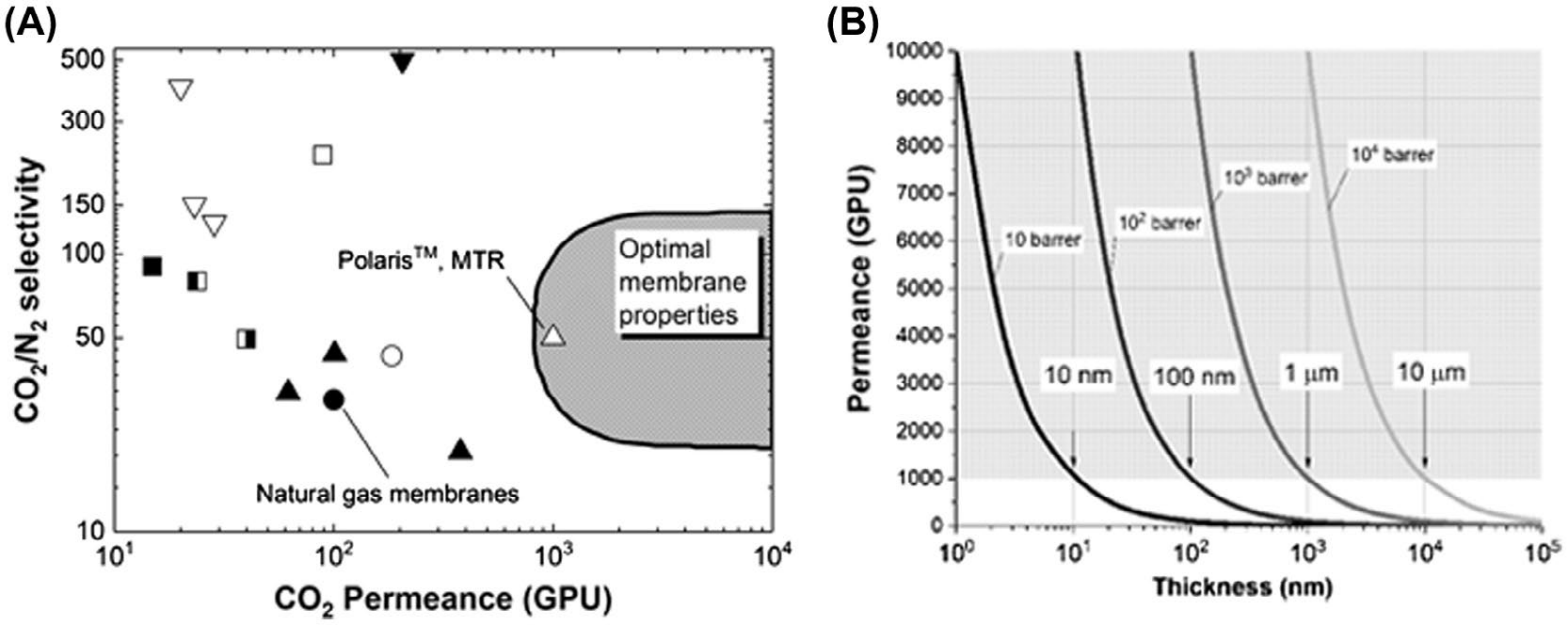
(a) CO_2_/N_2_ selectivity versus CO_2_ permeance trade-off plot comparing the performance of the MTR Polaris^TM^ membrane with commercial natural gas membranes and various developmental membranes reported in the literature. The shaded area shows the region of the optimum membrane properties for the separation of CO_2_ from a flue gas. The figure is reproduced from Merkel et al. [10]. (b) Relation between the permeance and thickness of the membrane showing what the appropriate thickness/permeability combination can potentially provide the membranes with sufficient permeances (gray area *P*>1000 GPU).

As estimated in a seminal work by Merkel et al. [10], in order to be economically feasible for industrial CO_2_/N_2_ separation, a membrane should possess a certain combination of selectivity and permeance, as shown in Figure 1(a). The Polaris^TM^ membrane developed by MTR, USA is one of the first examples that has reached the region of optimal properties for large-scale applications [10]. To reduce the cost, the values of the membrane permeance are dominant, and the CO_2_ selectivity of the membranes is a less critical factor. There are a few membrane examples that have reached permeances that exceed the 1000 GPU threshold. From Equation 1, the gas flux increases while decreasing the membrane thickness. The permeability of a membrane material is also an important factor; larger permeabilities enable higher gas fluxes at a fixed membrane thickness. Even if a membrane material has a sufficiently high gas permeability, thinner membranes are needed to achieve high gas permeances, as shown in Figure 1(b). Thus, membrane thinning can directly contribute to enhancing the gas permeance and can provide many opportunities to employ materials that do not have sufficient gas permeability as gas separation membranes.

 Nanomembranes are a class of objects with a thickness of less than 100 nm, and they possess large lateral dimensions which are usually several orders of magnitude higher than their thickness. With these unique features, nanomembranes can be manipulated without the use of special equipment. Most commonly, nanomembranes are made of organic polymers, however all different types of organic, inorganic, and hybrid materials could be utilized for fabrication. Membranes with a thickness of a few tens of nanometers (nanomembranes) can be prepared using various methods, including solution casting [13], dip and spin coating [14–17], layer-by-layer assembly [18], graphene oxide-polyelectrolyte assembly [19], and block copolymer self-assembly [20]; however, fabricating and handling membranes with thicknesses below 100 nm remains a challenging task. Membranes should be essentially free from physical defects, especially for gas separation, since defects as low as 10^−4^ can completely hinder the membrane separation ability [21]. In this review, we attempt to summarize the recent developments in supported nanomembranes used for CO_2_/N_2_ separation as well as the potential of freestanding nanomembranes.

## 2. Nanomembranes for CO_2_ capture

### 2.1. Nanomembranes on a highly permeable support

**Figure 2.**
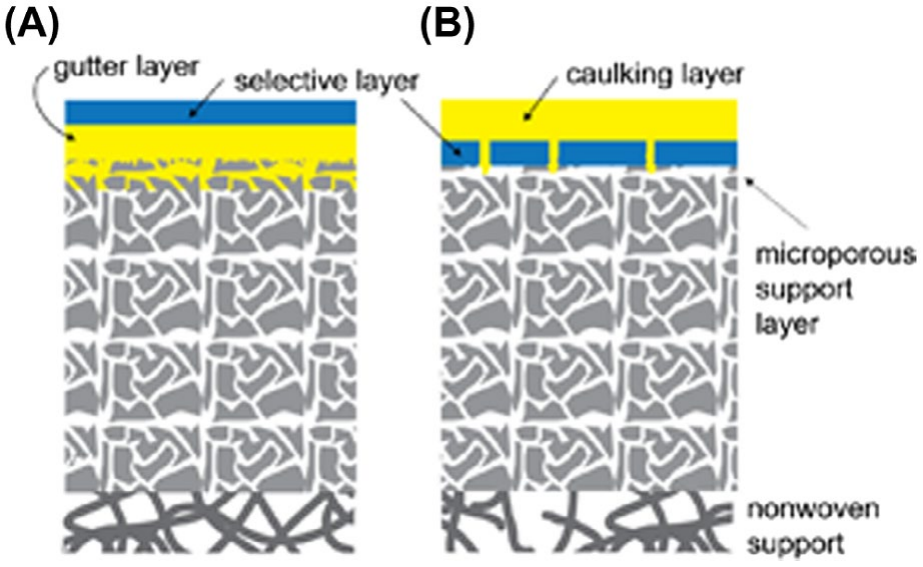
Schematic illustration of thin film composite (TFC) membranes. (a) A high permeability gutter layer is needed to smooth the porous support and prevent the formation of direct air gaps that may originate from the defects in the selective layer. (b) A high gas permeability caulking layer deposited on the surface of a skin layer formed in an asymmetric porous membrane is needed to fill the intrinsically formed defects.

Reducing the membrane thickness often causes defect formation, leading to unfavorable gas leakage. The defect problem can be partially solved by using so-called ‘gutter’ or ‘caulking’ layers. In these approaches, as shown schematically in Figure 2, the selective nanomembrane is placed or deposited on a completely defect-free and highly permeable support (gutter layer) or is alternatively coated with a similar material (caulking layer) that is believed to have negligible resistance to the gas permeation.

A caulking layer is used to coat a selective layer to fill its defects to avoid unfavorable gas leakage. For example, asymmetric polysulfone membranes have a surface skin layer that is formed during film preparation [10], and intrinsically, it contains a small number of defects that may lead to gas leaks. Depositing a highly permeable polymer with the main purpose of stopping the leaks through the defects usually results in restoring the selectivity of the target material.

In contrast, a gutter layer is first prepared on a porous support, following which a selective skin layer is formed on it. The utilization of a gutter layer is a much more common approach for the fabrication of thin selective layers, and polydimethylsiloxane (PDMS) is mostly utilized for this purpose. The role of a gutter layer is to prevent the precursor solution of the membrane material from penetrating into the porous substrate and blocking its pores. Additionally, a gutter layer provides a smooth and flattened surface and thus facilitates deposition of thin and defect-free selective layers [22]. Indeed, the utilization of highly permeable gutter layers on porous supports is a major approach that enables the formation of very thin selective layers for gas separation. For instance, the Polaris^TM^ membrane, which is a current benchmark produced by Membrane Technology and Research Inc. (Newark, CA, USA), also contains a gutter layer that is 50–200 nm thick, on which a selective layer (50–200 nm) is deposited by dip coating [23].

**Figure 3.**
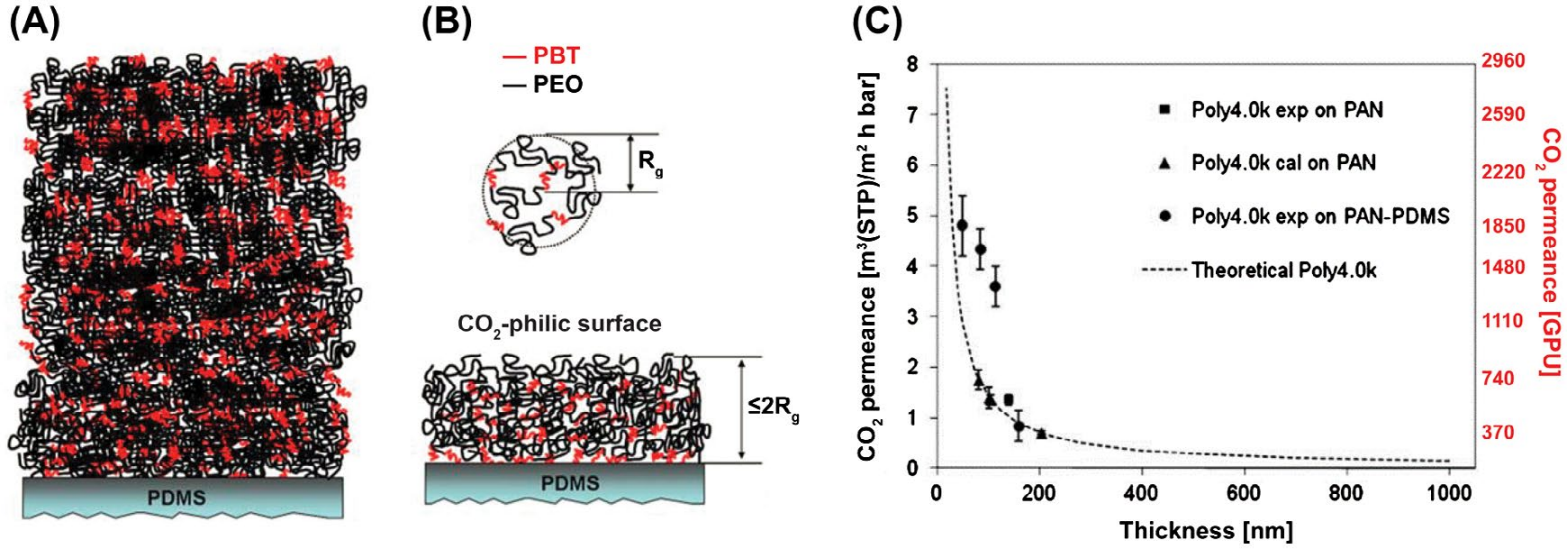
(a) Schematic representation of the block copolymer organization in thick films (semicrystalline polymer and high T_g_). (b) Representation of a polymer chain and ‘R_g_’ and the block copolymer organization within an ultrathin film under the influence of a PDMS substrate (a mostly amorphous thin film with a high fractional free volume and low T_g_ in the PEO segments), PBT stands for poly(butylene terephthalate). (c) CO_2_ permeance measured at 30 °C vs the thickness of the polymer thin film fabricated from the tailor-made Poly4.0k polymer. The experimental CO_2_ permeance values for the Poly4.0k thin films are two-fold higher than the calculated values (permeances calculated by using Equation (2) with a CO_2_ permeability value of 53 barrer for Poly4.0k). *Permeance scale with GPU units (red color) added to the original image for convenient comparisons with other data. Reprinted with permission from Yave et al. [24]. Copyright 2011 Royal Society of Chemistry.

Yave et al. [24] reported an improvement in membrane permeance by thinning selective layers from the submicron scale to a few tens of nanometers. They used a CO_2_-philic and tailor-made multiblock copolymer poly(butylene terephthalate)-b-poly(ethylene oxide) (PBT-b-PEO) with a controlled structure and molecular weight as a selective layer that was prepared on PDMS-coated PAN (polyacrylonitrile) supports. The researchers made a connection between the polymer radius of gyration (R_g_) and the dramatic permeance increase when the membrane thickness became comparable with the R_g_. Figure 3 shows the schematic of the block copolymer thin film deposited on the PDMS support. It was suggested that the organization of the copolymers changed when the thickness became similar to the radius of gyration (Figure 3(b)). The gas permeance of the membrane was nearly twice as high as the expected permeance calculated by using Equation 2 (Figure 3(c)). The reason for this behavior was also related to the alteration of the thermal properties of the material compared to those of the bulk system, as observed by nanocalorimetry. Namely, the PEO segments of the copolymer essentially remained in the amorphous phase in the ultrathin films and did not tend to crystallize, increasing the gas barrier as a consequence. As a result, the best membranes achieved CO_2_ permeances of 1300–1800 GPU with a selectivity that exceeded 50, which meets the requirements for CO_2_ capture applications formulated by Merkel et al*.* [10].

**Figure 4.**
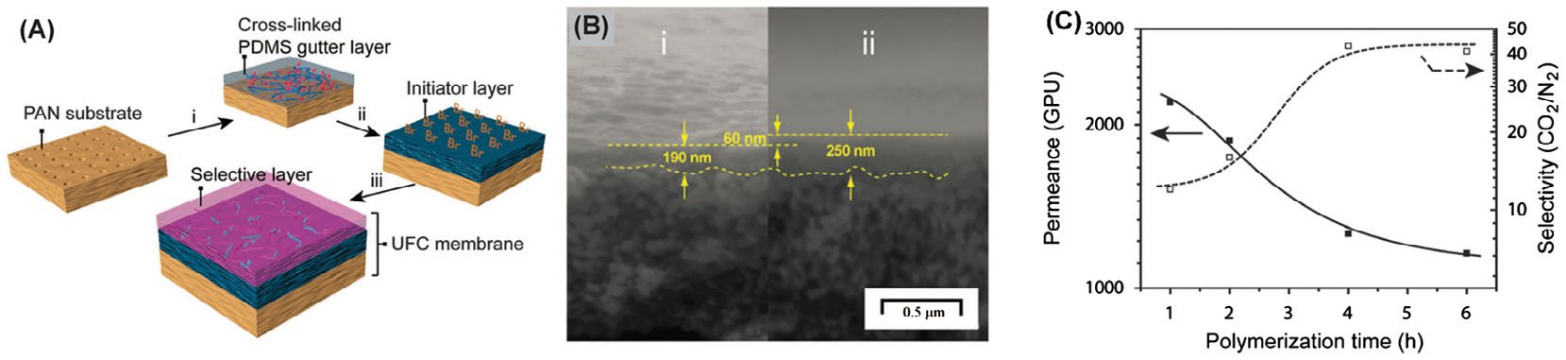
(a) Schematic illustration of the UFC membrane formation: (i) spin coating P2 (amino-terminated PDMS)/TMC to prepare the PDMS gutter layer; (ii) spin coating P3 (bromo-functionalized PDMS)/TMC to prepare the initiator layer; and (iii) CAP in the presence of CuBr_2_/Me_6_TREN/Na-ascorbate/crosslinkers (XLs) to afford the UFC membranes. (b) Scanning electron microscopy (SEM) images of the cross section of (i) the PDMS initiator layer and (ii) the UFC membranes (UFC1–6 h). The interface between the CAP film and intermediate PDMS layer cannot be distinguished, but by superimposing images of the PDMS initiator layer and multilayer UFC membranes, the thickness of the top layer can be estimated to be ca. 60 nm. The scale bar represents 500 nm. (c) CO_2_ permeance (solid symbols) and CO_2_/N_2_ selectivity (open symbols) through UFC3 series membranes as a function of the polymerization time. Reprinted with permission from Fu et al. [25]. Copyright 2015 Royal Society of Chemistry.

Fu et al. [25] recently developed a technology to achieve the continuous assembly of polymers (CAP) to prepare selective nanolayers with thicknesses from 40 to 140 nm on substrates. A general approach of CAP technology to fabricate ultrathin film composites (UFCs) is illustrated in Figure 4(a). A polyacrylonitrile (PAN) microporous substrate is employed as the support layer to provide sufficient mechanical strength. First, a solution of amino-terminated PDMS and 1,3,5-benzenetricarbonyl trichloride (TMC) is spin-coated onto the PAN substrate (Figure 4(a(i))), followed by introducing a bromo-functionalized PDMS precursor (Figure 4(a(ii))) as the initiating layer (Figure 4(a(iii))). Finally, the CAP of polyethylene glycol (PEG) macrocrosslinkers is achieved by utilizing atom transfer radical polymerization (ATRP) in an aqueous solution. This single-step CAP process, via propagating radical species transferred through the CAP film, results in UFC membranes with defect-free, surface-confined, and crosslinked ultrathin top layers [25].

CAP technology allows one to fabricate very thin selective layers (below 100 nm) as shown in Figure 4(b). As a result, TFC membranes fabricated by this approach exhibit very promising performance towards CO_2_ and N_2_ separation. As seen from Figure 4(c), longer surface polymerization times lead to thicker (less defective) but more selective and, at the same time, less permeable nanolayers. Due to their relatively slow growth, precise thickness control is possible by controlling the deposition time. Therefore, membranes with optimal permselectivity can be fabricated. CAP nanotechnology enables the preparation of different types of TFC membranes with mixed matrix materials as the selective layer, containing surface-functionalized SiO_2_ nanoparticles [26] or iron dopamine nanoparticles [27]. Although CAP is a new technology with only a few reported applications for gas separation, it provides new opportunities for CO_2_ capture, since the membranes prepared by CAP have exhibited some of the highest permeance/selectivity combinations to date [25–27]. In addition, CAP is based on a solvent process for membrane preparation, and it is advantageous for scalable and robust membrane fabrication.

In organic polymer membranes, significant numbers of studies have also reported the phenomenon of permeability decreasing with time, something attributed to the physical aging of the materials. Especially in thin films, the rate of permeability decrease is generally higher than that of rather thick films, and this phenomenon is common for glassy polymers. In glassy polymers, the aging effect on the gas permeability has been extensively investigated by Freeman et al. [28–30]. According to their interpretation, physical aging persists in glassy films and becomes significant when the thickness approaches the length scale of the individual polymer coils [28]. In particular, some films composed of polysulfone (PSF) and polyimide (Matrimid) with thicknesses of 18–550 nm exhibited reduced gas permeability of more than 50% after 1000 hours of aging at 35 °C with an increase in selectivity. The permeability of ultrathin films of glassy polymers can deviate dramatically from that of the bulk polymers, and the nature of these deviations is consistent with the enhanced mobility of the polymer chains and reduced T_g_ in ultrathin films [28].

**Figure 5.**
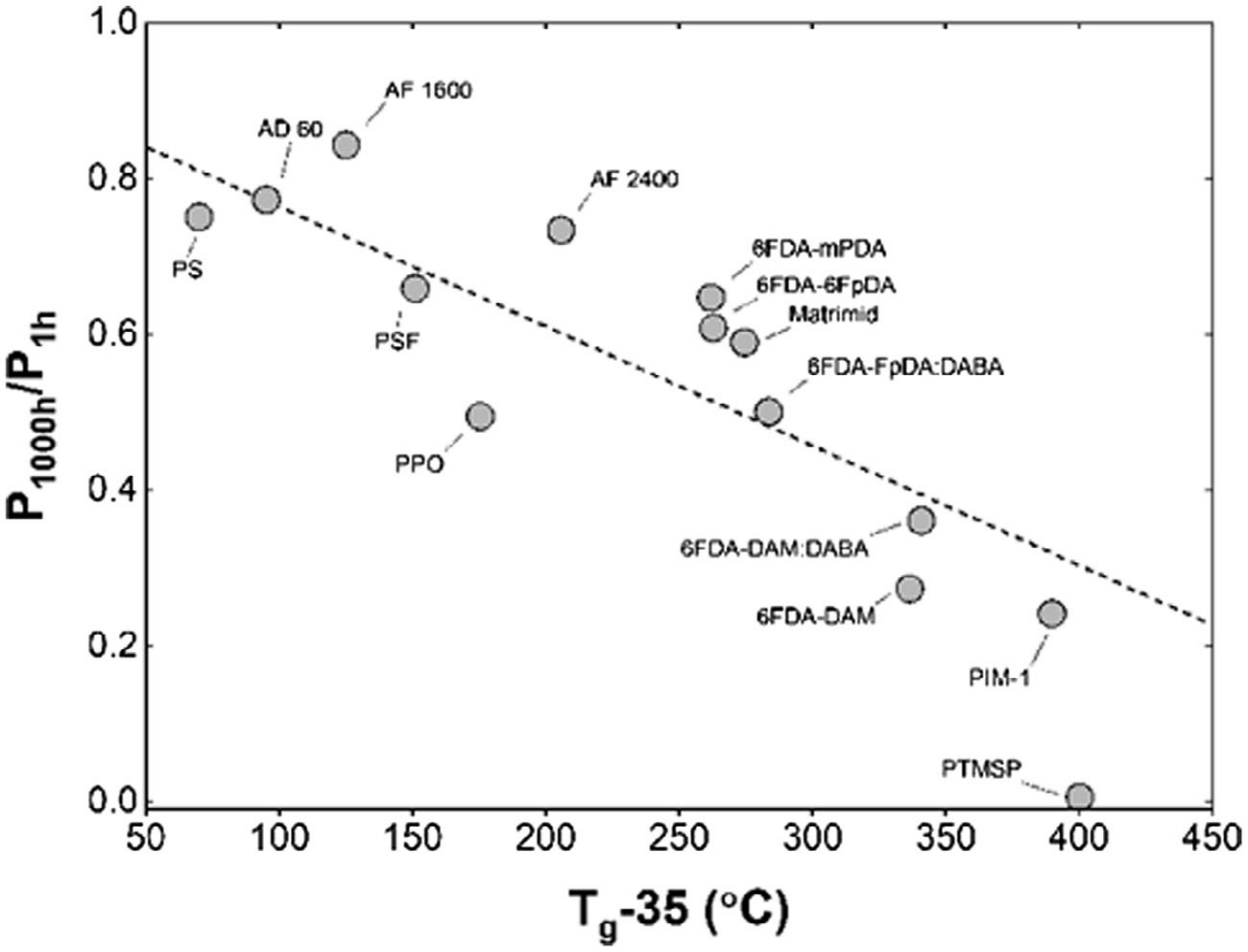
Correlation between the O_2_ permeability reduction rates with (Tg-35) (ºC) for glassy polymers with a thickness of ~400 nm or less, except for PTMSP, which had a film thickness ~700 nm. Reproduced from Tiwari et al. [29].

Similar studies were reported for other glassy polymers and, finally, the accelerated aging effect in thin films has been well correlated with the glass transition temperature of polymers rather than fractional free volume of polymers [29] (Figure 5).

Polymers with an intrinsic microporosity are promising materials for developing highly gas-permeable membranes. Among them, PIM-1 is the mostly investigated polymer for gas separation, and its thin films have also exhibited accelerated aging, which can lower the initial permeability by an order of magnitude. The relative permeability of thin films of PIM-1 (~220 nm) decreased by 67% compared to the 53% decrease for thick films (~30 μm) after 1000 hours of aging due to the higher aging rates of the thin films [30]. Thus, thinning the membranes of glassy polymers may not simply increase the gas permeance performance because of this accelerated physical aging phenomenon.

Several groups are searching for approaches to prevent physical aging in polymers. The aging of thick (~100 μm) films of super glassy polymers PIM-1, PTMSP, and PMP (poly(4-methyl-2-pentyne)) was successfully prevented by adding microporous microparticles of porous aromatic framework (PAF-1, tetrakis(4-bromophenyl)-methane) [31]. In their most recent work, the same group reported that aging could be significantly suppressed in much thinner PTMSP films (~1 μm), again by introducing PAF-1 or a hypercrosslinked polymer, such as polydichloroxylene (p-DCX) [32]. However, there is still a lack of published works in the field of aging prevention, and practically none of these studies have dealt with the aging of ultrathin films, to the best of our knowledge.

In contrast, the selectivity usually increases in thin films during aging; therefore, one could consider using aged membranes beneficially. However, to the best of our knowledge, the aging process is still considered to be a strong disadvantage for industrial applications, requiring rather stable and predictable materials. In particular, the very rapid aging observed in polymers with an intrinsic microporosity is a barrier that prevents their use in industry.

Conversely, aging in rubbery polymers may not be an issue compared to glassy polymers, since their T_g_ always lies below the working temperature. In earlier work, the oxygen permeability of a PDMS membrane with a thickness of 0.61 μm was stable and independent of the aging time [33]. Recently, Firpo et al. [34] investigated the influence of thickness on the permeability of the PDMS membranes in more detail. Surprisingly, a significant decrease in permeability was observed even in the membranes with thicknesses below tens of micrometers. This was most likely an effect of the oxygen plasma treatment that enhanced the gas barrier properties of the investigated films. Authors used the numerical model developed by Islam and Buschatz [35] that is based on a nonequilibrium sorption–desorption process at the membrane interface.

In general, the physical properties of polymeric thin membranes become different from those of the bulk film, and these differences can alter gas permeance behavior. Many different physical parameters in thin films have also been reported, for instance, the Young’s modulus [36] and wettability [37] increase in Nafion films with thicknesses below 100 nm, although the glass transition temperature (T_g_) decreases in ultrathin films [38,39]. It is generally known that the gas permeability of bulk polymers inversely correlates with the T_g_ [40], i.e. polymers with higher T_g_ values are expected to have a smaller permeability. According to this knowledge, reducing the T_g_ in ultrathin membranes is expected to increase gas permeability. However, for most polymers in thin films, permeability shows the opposite behavior. Therefore, it would not be sufficient to discuss the gas permeability of thin membranes only in terms of the thermal properties of polymeric membranes, and there is still a lack of systematic research that has investigated the phenomenon of the permeability deviating from the bulk state at ultimate thicknesses; this research area deserves further study.

The difficulties of developing ultimately thin membranes using ordinary organic polymers by conventional methods have caused researchers to focus on using intrinsically thin 2D materials, such as graphene, graphene oxide and their inorganic analogs, hexagonal boron nitride (h-BN) and transition metal chalcogenides (e.g. MoS_2_, WS_2_) [41]. The expected advantages of such 2D materials include their well-ordered and stable molecular network structures compared to conventional organic polymer chains, enabling gas permeation channels to be designed more precisely.

Graphene and graphene oxide (GO) are by far the most prevalent materials for fabricating various membranes. For instance, Kim et al. [42] reported selective gas transport through few-layer graphene oxide membranes coated on highly gas permeable microporous polyethersulfone supports. They found that gas permeation is highly dependent on the method of GO layers assembly. GO membranes formed highly interlocked layer structures and exhibited extraordinary gas permeation behavior with high carbon dioxide/nitrogen selectivity (up to 20) when maintained in the hydrated state, which strongly inhibited nitrogen permeation. This work revealed the possibility of achieving selective gas transport through ultrathin (~3 to 7 nm), few-layer GO membranes [42].

**Figure 6.**
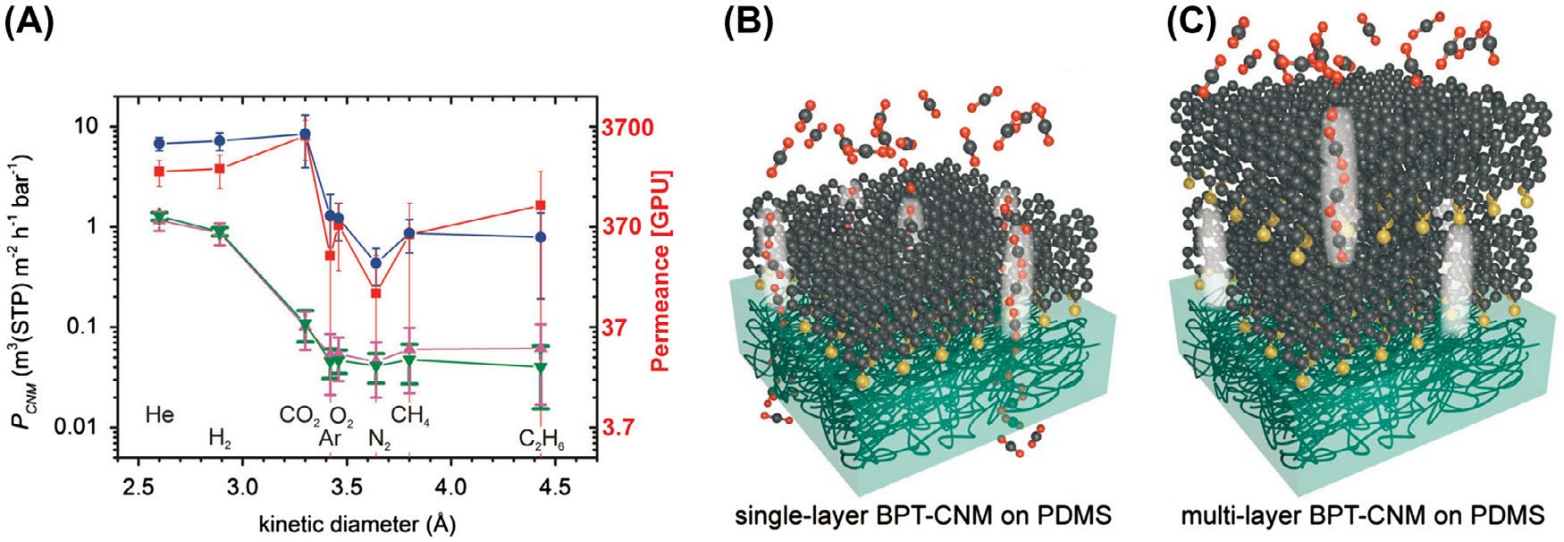
(a) Gas permeance through different CNM membranes (*P*_CNM_) calculated from the permeance of the composite membranes (CNM/PDMS) using resistance models that account for the finite permeance of the PDMS support and partial coverage of the PDMS surface by the CNM. * Permeance scale with GPU units (red color) added to the original image for convenient comparison with other data. (b) Schematic depiction of the proposed gas transport mechanism in single and multilayer CNMs. The molecule-sized channels (highlighted by the bright regions) favor the permeation of CO_2_ and smaller gas molecules. (c) Low diffusion of CO_2_ in between the individual CNMs hinders its permeation through the multilayer CNMs**.** Reprinted with permission from Ai et al. [43] Copyright 2015 WILEY-VCH Verlag GmbH & Co. KGaA, Weinheim.

Carbon nanomembranes (CNMs) are a new class of 2D carbon materials with possible thicknesses below 2 nm. In this approach, the membrane is fabricated by forming highly ordered self-assembled monolayers (SAMs) on solid substrates followed by exposure to an electron beam or extreme ultraviolet light in order to crosslink the molecules within the SAM. The resulting freestanding nanolayer with a thickness below 2 nm can be transferred on a different support without losing its integrity. Ai et al. [43] attempted to measure the gas permeance properties of one- and three-layer CNMs transferred onto a thin PDMS support. A series resistance model [21] was employed to estimate the intrinsic properties of the CNM gas permeation. Figure 6 shows the calculated permeance results obtained for the one- and three-layer CNMs prepared using two different SAM precursors – 1,1′-biphenyl-4-thiol (BPT) and 4′-nitro-1,1′-biphenyl-4-thiol (NBPT). Comparing the results of these membranes with the permeance of the support PDMS layer alone demonstrated that the CNMs exhibited increased selectivity for smaller molecules over larger ones (Figure 6(a)). For the case of CO_2_, the authors suggested the existence of molecular-sized channels in the 1-nm-thick membrane were favorable for CO_2_ transport (Figure 6(b)). However, transmembrane channels were not formed because of the unmatched positions of the individual channels between the three layers of the CNM (Figure 6(c)), similar to previous observations of graphene-based membranes [42]. In terms of the permeance, despite its ultimate thickness, the CNM exhibited very low permeances, namely, ca. 300 and 40 GPU for CO_2_ using one-layer and three-layer CNM, respectively. Future major advancements in CNM molecular design will be crucial in order to enhance the gas separation ability.

In all the above cases, a highly gas permeable underlayer, such as PDMS, is used as a gutter layer on the porous substrate. This tri-layered structure (selective layer/gutter layer/porous support) provides several advantages especially for selective layers, such as requiring a smaller amount of material for fabrication and not requiring significant mechanical robustness as a freestanding film. Therefore, this approach has been widely used commercially and allows one to produce selective layers with a thickness of less than 100 nm.

**Figure 7.**
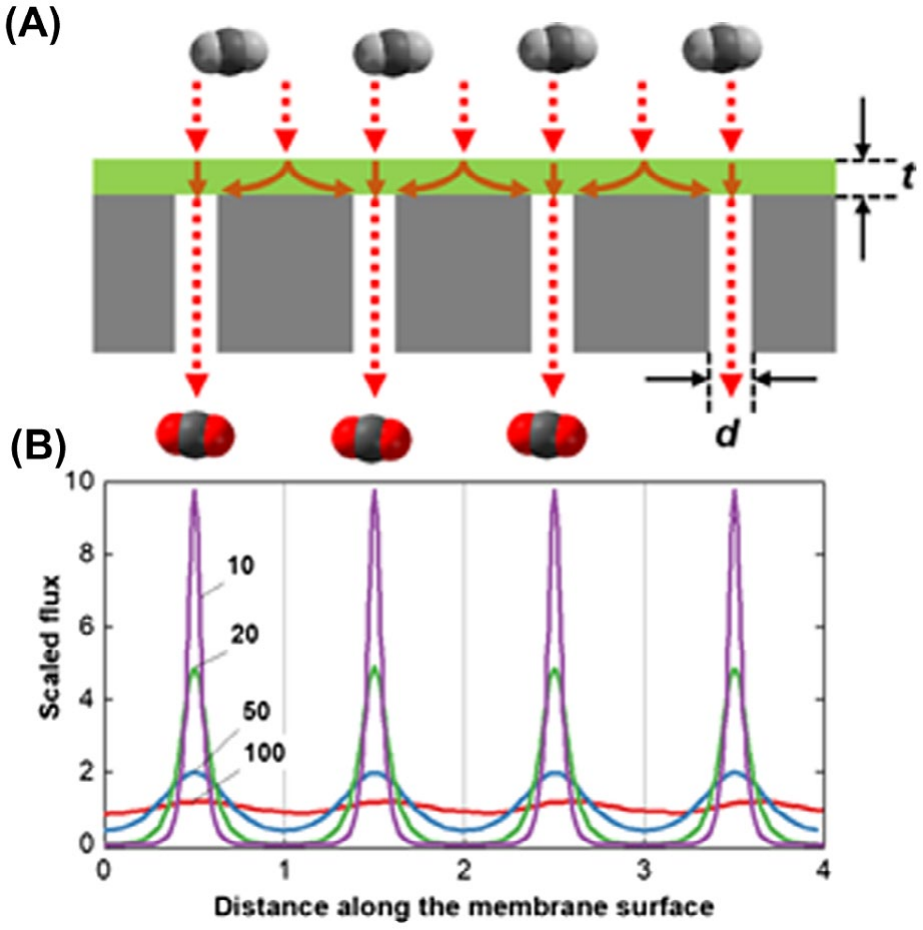
(a) Schematic illustration of the gas flux through the membrane (green) and porous support (gray). The membrane thickness and pore diameter of the porous support are *t* and *d*, respectively. (b) The scaled flux shown for various values of the scaled thickness, *H* = *d*/*t,* reproduced from Ramon et al. [46].

However, simply thinning the selective layer in this tri-layer structure may not be sufficient for enhancing the overall membrane performance [44–46]. Figure 7(a) shows a schematic illustration of the gas permeance across the membrane (the green part, *t*, indicates the membrane thickness, including a selective and gutter layer) and the porous support (the gray part, *d*, indicates the pore diameter). Gas molecules must reach the pores in the porous support after passing through the membrane. The supports have open pores that are separated by a certain distance. Figure 7(b) shows the scaled gas flux through the membrane along the membrane surface position; the flux depends on *H*, which is the ratio of the membrane thickness to the support pore radius [46]. In thicker membranes (*H* = 100), the gas flux across the membrane is practically equal at any point on the membrane. However, further membrane thinning (*H*<50) causes a nonuniform distribution of the gas fluxes. The gas flow at the interface of the open pores and the porous support becomes dominant, and it becomes negligibly small in the places without open pores. Although thicker gutter layers may mitigate this geometric restriction of the porous support on the gas permeance, thinner gutter layers also face a similar situation. The minimum thickness of the gutter layer was estimated to be one or two times larger than the diameter of the open pores in the support [45].

Considering the geometrical limitations of classical composite membranes, new approaches to the problem of thinning are needed. We believe that the utilization of mechanically strong freestanding nanomembranes could be a solution, in which thinning is only limited by the material, and the influence of the microporous support could be minimized.

### 2.2. Freestanding nanomembranes

Freestanding nanomembranes have the potential to avoid the drawbacks of the support layer and have the following three features: their thickness is in the range of 1–100 nm; they have self-supporting properties [47,48]; and they have aspect ratios of size to thickness greater than 10^6^ [47]. To use a freestanding membrane for gas separation, nanomembranes should have sufficient mechanical robustness to hold their membrane shape and must not have any defects at the molecular level to avoid unfavorable gas leakage. Of course, large-scale processability for membrane production is also an important factor for enabling industrial use.

Graphene is an atomically thick 2D material of sp^2^-bonded carbon atoms and is one of the thinnest materials. Recently, the large-scale preparation of graphene sheets has been reported by Li et al. [49–51], and this thinnest carbon-based ‘nanomembrane’ can thus be considered as a platform for separation [52] because defect-free graphene acts as a completely gas impermeable material [53]. Bunch et al. [53] employed a laser ablation process to introduce molecule-sized pores in single-layer graphene and reported the phenomena of preferential gas permeation through the designed pores. However, this approach is far from the mass production of graphene-based separation membranes. The creation (introduction) of well-engineered nanopores in large-area graphene membranes still remains a challenging issue to be used effectively for gas separation.

Graphene oxide (GO) has also been extensively studied for gas separation. GO is also a two-dimensional carbon sheet with various oxygen-containing functional groups on its basal planes and at its edges. Li et al. [54] reported the molecular sieving effect of GO for selective hydrogen separation. In their approach, anodized aluminum oxide (AAO) was used as a support instead of using a nonporous and highly gas permeable polymer substrate. AAO clearly exhibited pores at its surface, and the GO sheets covered over the pores completely without any intermediate layer. From this viewpoint, the GO sheets acted as a freestanding membrane. In their report, the thickness effect of the GO membranes on gas selectivity was discussed, and highly H_2_-selective separation was achieved based on the molecular sieving effect. During the separation of CO_2_ over N_2_, molecular sieving can also be utilized upon achieving precise pore control. However, due to the small kinetic diameter difference between the molecules, this approach faces the same issue: controlling the pore size and CO_2_ affinity at the edges of the pores.

Although graphene or graphene oxide are very attractive materials for separation membranes, the ability to design the materials and chemically tune the CO_2_ affinity is still limited. There have been many efforts to fabricate freestanding nanomembranes with larger lateral sizes (more than the centimeter scale) using conventional film preparation technologies, such as spin and dip coating, interfacial polymerization, and roll-to-roll processes. In these approaches, the thickness of the membranes has not yet reached the atomic scale and remains in the range of a few tens of nanometers (or less). However, the major advantage of these approaches, compared to the use of graphene or other 2D materials, is the wider availability of materials.

Kunitake et al. [14,15,47,55] succeeded in preparing freestanding and large nanomembranes using polymers, metal oxides, and their composites. In their approach, the membranes were prepared by using the spin coating process, which is widely used in industry. The utilization of a sacrificial layer is essential for nanomembrane detachment. A huge number of material combinations are available for nanomembrane fabrication using this approach. For example, interpenetrating molecular networks of organic polymers and ZrO_2_ in freestanding nanomembranes (approximately 35 nm thick) demonstrated high mechanical robustness; the membrane was able to hold an amount of liquid that was 70,000 times heavier than its own weight without any under support [14]. The mechanical strength of these freestanding nanomembranes is an undoubtful advantage for separation membranes.

Various post treatments may be applied for freestanding nanomembranes to improve their properties. For instance, Mallwitz and Goedel [56] used physical crosslinking instead of chemical crosslinking in order to add more flexibility to covalently crosslinked Langmuir-Blodgett monolayers. As a result, monolayers of hydrophobic polymers with a low glass transition provided elastomeric freely suspended membranes that were intact after drying and one year of storage.

Simple modifications of membrane matrices may be used to introduce different functions and improve molecular selectivity [57]. For instance, for precise molecular separation, Fujikawa et al. [58] reported nanochannel formation in a freestanding nanomembrane by molecular imprinting. Amorphous titanium oxide was used as a membrane matrix, and the nanochannels prepared from the template molecules precisely recognized the molecular size, resulting in preferential molecular separation by conventional vacuum filtration. In this case, certain amounts of the template molecules added to the membrane material precursor were necessary for the formation of nanochannels. Overlapping or neighboring among the template molecules in a membrane matrix led to the formation of continuous pores after templates removal.

A similar approach was employed to achieve preferential CO_2_ separation. Selyanchyn et al. [59] reported the incorporation of CO_2_-philic molecules (phthalic acid) into titania nanolayers. Such nanolayers assembled on a PDMS support demonstrated high CO_2_ selectivity over nitrogen, although practically no inherent selectivity was observed without incorporating the CO_2_-philic molecules. Unfortunately, this nanolayer did not have sufficient mechanical stability as a freestanding membrane, but this result supports this molecular design concept; the incorporation of molecular sites with a chemical or physical affinity to target molecules within a nanomembrane can work well for preferential gas separation.

Similarly, the design of transmembrane functions in proton-conductive membranes has also been reported [60]. In the reported case, percolated connections of Brønsted acid centers played an important role in achieving efficient proton conduction. When the maximum cluster size of these neighboring Brønsted acid centers was similar to the membrane thickness, proton percolation became possible. In contrast, when the membrane thickness was much larger than the maximum cluster size, smooth proton conduction was not observed. Although this is the explanation for unusual proton conduction in nanometer-thick membranes, we could find intrinsic similarities between the gas and ion transport across the membranes. It would be difficult to reproduce similar permeation phenomena in thicker membranes because the clusters composed of neighboring affinity sites should become bigger, leading to less selectivity or more complex membrane processability. In addition, much longer gas permeation channel lengths would increase the gas permeation resistance, resulting in a significant permeability decrease until becoming nearly impermeable. Therefore, nanomembranes present the only option for designing selective molecular channels without losing feasible permeability. In a further step, these nanomembranes could be transferred onto a macroporous support, indicating that membrane preparation and setting the membranes on porous supports can be independent processes. Due to their intrinsic strength, the employment of freestanding nanomembranes for gas separation allows one to use a variety of porous supports for performance enhancement.

**Figure 8.**
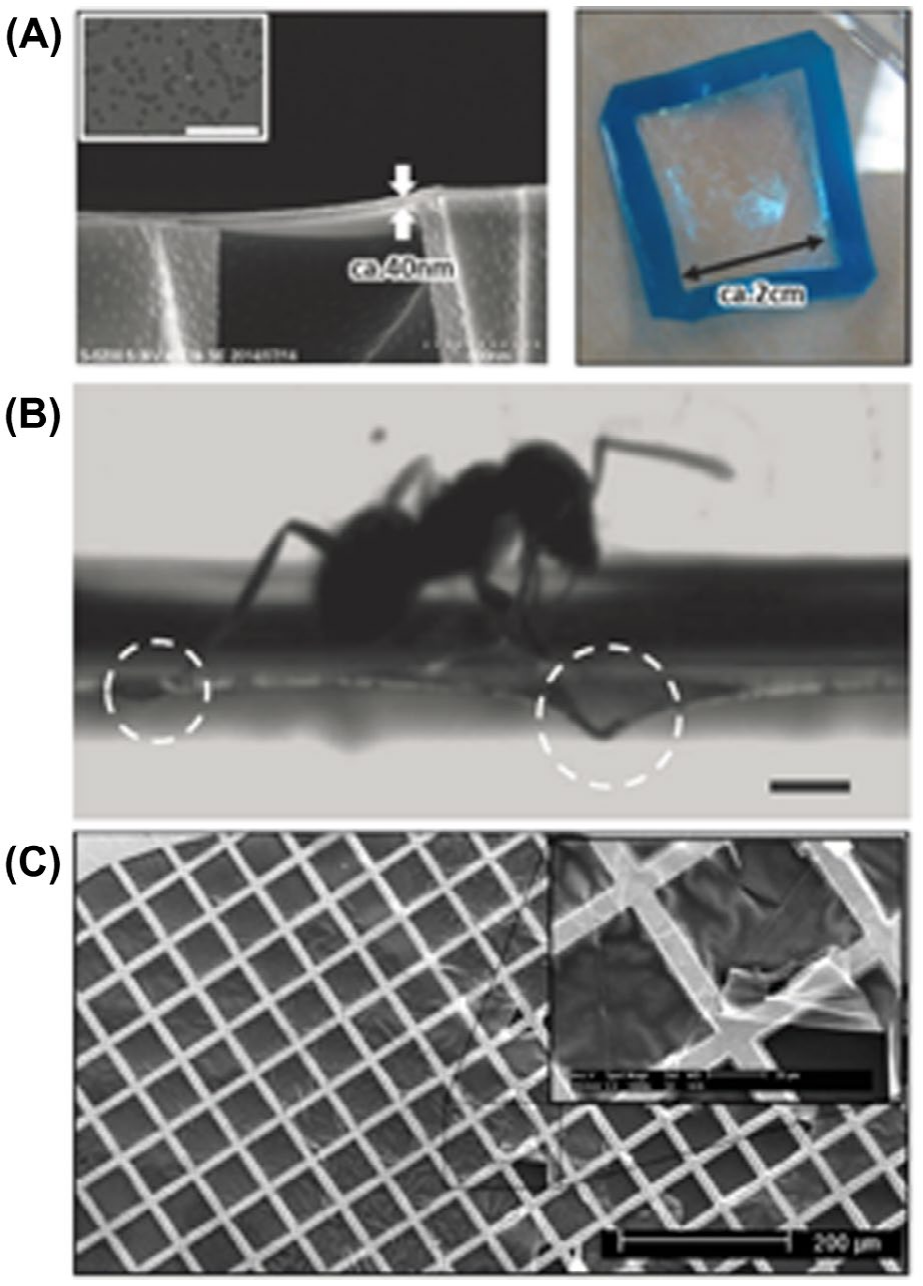
(a) SEM (left) and photography (right) of robust PEI/PCGF nanomembranes with a thickness of ~40 nm on a porous polycarbonate support and in freestanding condition respectively. Reprinted with permission from Fujikawa et al. [61]. (b) Photograph of an ant walking on a PDMS membrane with a thickness of ca. 90 nm. Reprinted with permission from Kang et al. [62] Copyright 2013 WILEY-VCH Verlag GmbH & Co. KGaA, Weinheim. (c) SEM image of a 10 nm thick CMP nanomembrane transferred onto a transmission electron microscopy grid. Reprinted with permission from Lindemann et al. [66] Copyright 2014 American Chemical Society.

The primitive use of freestanding nanomembranes for CO_2_ separation was reported in several works. For example, crosslinkable polymer materials, conventional epoxy resin (poly[(o-cresyl glycidyl ether)-co-formaldehyde], PCGF), and branched poly(ethyleneimine) (PEI) were used to fabricate freestanding PEI/PCGF nanomembranes with a thickness down to ca. 20 nm. By simply spin-coating a component mixture, nanometer-thick epoxy films were fabricated and detached as freestanding membranes (Figure 8(b)). In this case, epoxy groups reacted with the primary or secondary amines in PEI and formed ethanol-amine structures that could act as CO_2_ affinity sites. As-prepared nanomembranes showed moderate CO_2_ selectivity over N_2_ under humid conditions [61]. Although more sophisticated membrane material designs are necessary, this result indicates that the as-prepared nanomembranes did not possess serious defects that led to simple gas leakage. That result demonstrates an example of how nanomembranes can be utilized for CO_2_ separation with a potentially high gas flux.

Several other groups have developed nanomembranes made of different materials. For example, Kang et al. [62] demonstrated freestanding nanomembranes composed of PDMS with thicknesses reaching 77 nm. Although the gas separation properties of the nanomembranes were not tested in their work, the mechanical properties of the membranes were confirmed by liquid weight support without membrane wrapping (e.g. Figure 8(a) shows an ant supported by a ca. 90 nm thick membrane). Considering the moderate selectivity towards CO_2_ (α~11.6 at room temperature) and the high gas permeability of PDMS [63] such nanomembranes should provide permeances that far exceed the requirements for optimal industrial applications, while the selectivity requires optimization.

Meyerbröker et al. reported the formation of ultrathin films [64] and nanomembranes [65] based exclusively on crosslinked poly(ethylene glycol) (PEG). Freestanding membranes with thicknesses ranging from 10 to 300 nm were prepared by a simple procedure, and they possessed several useful properties, including hydrophilicity, biorepulsion, and extreme elasticity due to having a very low Young’s modulus of ca. 2 MPa [64]. The combination of elasticity and mechanical stability makes these membranes potentially useful as highly sensitive elements in microelectromechanical systems. Considering the high selectivity of PEG to CO_2_, these membranes may also be promising for gas separation tests, which have not been conducted to the best of our knowledge.

In another work, Lindemann et al. [66] succeeded in fabricating virtually defect-free nanomembranes with sub 10 nm thicknesses (Figure 8(c)) using the layer-by-layer growth of ‘click’-based conjugated microporous polymers (CMP) on sacrificial supports. For gas tests, however, membranes should be placed on a standard PDMS-coated PAN support, and the estimated permeability for hydrogen was only ~ 4 barrer, which was much less than expected for rigid porous materials. The gas separation results of this work are similar to those observed for CNM membranes. Unfortunately, despite their freestanding property, it was not possible to measure the gas separation without a PDMS gutter layer, which again suggests that glassy organic polymers are less likely to be utilized as materials for efficient nanomembranes.

## Conclusions

As demonstrated by the majority of the literature data, once reaching the ultimate thickness, membrane materials change their properties toward gas separation. The classical expectation for gas permeance improvement by decreasing the thickness of a membrane starts to deviate in the submicron range. Most materials exhibit a decrease in permeability. This phenomenon in glassy polymers has been attributed to physical aging. However, despite the fact that aging is not characteristic for rubbery polymers, they also show decreased permeability with a decreasing membrane thickness, for which, so far, there is no clear explanation. A few researchers have tried to explain the decrease in permeability by interfacial effects, while others have assumed different morphological features for the ultrathin materials. When using the most common tri-layer membrane assembly (porous support/gutter layer/selective layer), the influence of the support also becomes significant once the selective (gutter) layer becomes ultrathin. Therefore, new membrane architectures are critically needed in order to improve gas separation performance, especially the gas flux. Freestanding nanomembranes are one of the alternative approaches, although they are not yet well developed. Nanomembranes allow one to tune the gas affinity and ‘diffusion’ through the membranes at the molecular scale. Specifically, gas adsorption and desorption will be mainly governed by the surface properties, which can be more flexibly modified by post treatment or during preparation.

An example of an ideal separation membrane can be found in biological systems, namely, lipid bilayer membranes. From the viewpoint of membrane performance, biological membranes have a high selectivity and flux under ambient conditions due to their molecular thickness and self-supporting nature. This sophisticated example encourages further exploration in thinning synthetic membranes in an attempt to reproduce the superior performance achieved in nature.

In conclusion, we believe that membrane thinning for more efficient CO_2_ separation has high potential and is a critical area for research aiming to solve the problem of global warming.

## Funding

This work was supported by World Premier International Research Center Initiative (WPI), MEXT, Japan. In addition, the work was supported by a Grant-in-Aid for Scientific Research (S) [grant number 25220805] from the Ministry of Education, Culture, Sports, Science, and Technology (MEXT) of Japan and a JSPS Kakenhi Grant [grant number 16H06513]. We gratefully acknowledge financial support from JST ACT-C [grant number 24550126]. The work was also supported by the Japanese government (MEXT) scholarship program and by the Japanese Society for the Promotion of Science [JSPS Grant-in-aid for Research Activity Start-up, grant number 26889045].

## Disclosure statement

No potential conflict of interest was reported by the authors.
